# Retraction Note: Sulforaphane potentially attenuates arsenic-induced nephrotoxicity via the PI3K/Akt/Nrf2 pathway in albino Wistar rats

**DOI:** 10.1007/s11356-024-34526-w

**Published:** 2024-08-01

**Authors:** Shanmugam Thangapandiyan, Mathan Ramesh, Selvaraj Miltonprabu, Tamilselvan Hema, Gunasekaran Bavithra Jothi, Venkatesan Nandhini

**Affiliations:** 1 N-PDF, Unit of Toxicology, Department of Zoology, Bharathiar University, Coimbatore, Tamilnadu 641 046 India; 2 Unit of Toxicology, Department of Zoology, Bharathiar University, Coimbatore, Tamilnadu 641 046 India; 3 Department of Zoology, University of Madras, Chennai, Tamilnadu 600025 India

**Retraction Note: Environmental Science and Pollution Research (2019) 26:12247-12263**

10.1007/s11356-019-04502-w

The Editor-in-Chief and the publisher have retracted this article. Concerns have been raised regarding a number of figures, specifically:

• Figure 6a: gels appear to show signs of crop marks and bands appear to be to be visible multiple times within the same blots, or between blots

• Figure 7a: the gels appear to show signs of crop marks and bands appear to be visible multiple times within the same blots, or between blots

• Figure 8a: panels c and d appears to show an area of overlap

• Figure 9: panels a and c appear to be identical.

• Figure 9: panels b and c appear to be identical, but turned 180 degrees

• Figure 10: panels a and e appear to be identical, but turned 180 degrees

• Figure 11: all four panels show unexpected repeated patterns

• Figure 11: panels a and c appear to be similar, but mirrored

• Figure 11 also appears to have been previously published as Figure 14 in (Shanmugam et al. 2018)

The Editor-in-Chief therefore no longer has confidence in the reliability of the results and conclusions of this article.

The Authors have not responded to any correspondence from the publisher about this retraction.
